# Worldview, psychological flexibility, and depression-anxiety-stress in Chinese youth

**DOI:** 10.3389/fpsyg.2024.1447183

**Published:** 2024-12-12

**Authors:** Sheng-Li Cheng, Xin Zhang, Chenxu Zhao, Yun Li, Shushan Liu, Sanyin Cheng

**Affiliations:** ^1^School of Philosophy and Social Development, Shandong University, Jinan, China; ^2^Business School, Shandong Agriculture and Engineering University, Jinan, China

**Keywords:** depression, anxiety, stress, worldview, psychological flexibility

## Abstract

**Background:**

The psychological problems among the youth population have received widespread attention in the information age. However, little research has been conducted on the effects and mechanisms of worldview on depression, anxiety, and stress (DAS) among youth. In this study, we aim to investigate the relationship between worldview and DAS among youth.

**Aims:**

The aim of this study is to investigate the current state of worldview, psychological flexibility, and DAS in Chinese youth groups, and to explore the relationship between youth worldview, psychological flexibility, and DAS.

**Methods:**

A total of 2,351 Chinese youths completed questionnaires measuring their worldview, psychological flexibility, and DAS levels. The data were analyzed using structural equation modeling (SEM).

**Results:**

The impact of youth worldview on DAS was sophisticated. Positive worldview had a negative direct predictive effect on DAS, as well as a negative indirect predictive effect mediated by psychological flexibility. Traditional worldview had a positive direct predictive effect on DAS, a negative indirect predictive effect mediated by psychological flexibility, and a positive total predictive effect. Spontaneous worldview only had positive indirect predictive effects on DAS. Pessimistic worldview had positive direct effects on DAS and indirect effects mediated by psychological flexibility. Policymakers, psychologists, and educators working with youth should carefully consider the implications of these results for education, employment, and mental health.

## 1 Introduction

As defined by the United Nations (1985), “youth” includes individuals aged 15–24, a critical period of rapid personal development and identity formation (Bucholtz, 2002). This age group increasingly faces unique challenges in the digital era, as the proliferation of information online can overwhelm their developing judgment abilities, often leading to heightened stress, identity confusion, and mental problems such as depression and anxiety (Herman et al., 2021; Kuehn et al., 2023; Marshall et al., 2016). Against this background, Sue (1977) introduced the concept of worldview into psychotherapy, providing a new perspective for addressing mental health problems among youth. Worldview is an umbrella term for an individual’s fundamental beliefs and values about the world. It influences not only an individual’s cognition, emotions, and behaviors but also their mental health status (McLaughlin, 1993; Tonmyr and Blackstock, 2010). Additionally, numerous studies have confirmed that psychological flexibility plays a crucial mediating role in the examination of youth mental health (Li et al., 2022; Wang et al., 2023). Furthermore, enhancing the psychological flexibility of young people tends to alleviate the severity of mental health problems (Hayes et al., 2006; Li et al., 2015). Through extensive research on these issues, it may be possible to offer new theoretical support and practical guidance for preventing and intervening in youth mental health problems.

### 1.1 Relevant research on depression, anxiety, and stress in youth

In recent years, research on depression, anxiety, and stress (DAS) among youth has gained increasing attention due to the unique vulnerabilities of this age group (O'Neil et al., 2020; Sanchez Merino et al., 2023). Adolescence and early adulthood are marked by rapid psychological, social, and biological changes that can profoundly affect mental health, often resulting in decreased self-confidence, mood swings, and academic inefficiency (Khan et al., 2021; Pakhomova et al., 2021). These challenges can impact various aspects of the lives of youth, including their work, school performance, social relationship, and family dynamics (Kudryavtseva, 2021; Vadakkiniath, 2023), ultimately diminishing their quality of life and social functioning. Therefore, it is important to address their DAS. This can enhance their self-regulation ability and improve their quality of life, providing healthier and more stable human resources for society’s development (Armstrong, 2009; Hochi et al., 2020; Scavarda et al., 2019).

Current research on anxiety, depression, and stress among youth in the field of psychology mainly includes three areas. First of these is describing the current state of DAS among young people. Henry and Crawford (2005) used the DASS-21 to measure the mental health problems in the Spanish youth population, finding that the mean scores for depression (*M* = 6.29) and anxiety (*M* = 6.02) were lower than the theoretical median, while stress (*M* = 13.92) was above the theoretical median, reaching a concerning level. Zlomke (2009) measured DAS among American youth, with scores for depression and anxiety higher than those of Spanish youth, while stress was lower than in Spanish youth but still above the theoretical median. Research conducted by Gong et al. (2010) among 1,799 Chinese college students showed that DAS among Chinese youth was below the theoretical median, indicating better mental health conditions compared to youth in other regions of the world.

Secondly, studies have been conducted on DAS as triggers for other mental problems and as indicators in assessing psychotherapy outcomes. Bai et al. (2020) and Dwight et al. (2024) explored the correlation between psychological symptoms and parental mental health from the perspectives of both youth and clinicians. Through their study they aimed to evaluate the feasibility and effectiveness of using the DASS-21 as a tool for monitoring therapeutic progress.

Thirdly, the causes and optimization pathways of DAS in young people are examined. Social relationships in youth populations have a strong correlation with negative mood states. Gonzálvez et al. (2018) surveyed 1,582 Ecuadorian adolescents and found that higher levels of school rejection behaviors were associated with higher levels of negative mood. Rivas (2021) recruited participants aged 18–25 from across the United States. The data analysis revealed that youth at transition age who had low-quality relationships with their parents were more likely to endorse substance use. Research has shown that a positive youth worldview may reduce levels of DAS (Cheung et al., 2022; Kewley et al., 2018). Additionally, researchers have examined the impact of the internet on youth mental health. Studies suggest that social networks and online positive psychology programs can enhance youth well-being and mental health (Manicavasagar et al., 2014; Orsolini et al., 2022).

While existing research has made strides in understanding the current state and influencing factors of DAS among various youth groups, most studies emphasize individual and family dynamics. Research examining the cognitive and social dimensions that underlie these issues remains limited. In light of the observed increase in DAS among Chinese youth post-COVID-19 (Guo et al., 2022; Pakenham et al., 2024), it is critical to explore the cognitive factors that link individual experiences with societal influences to better understand and address the underlying determinants of DAS in this population.

### 1.2 Research related to the relationship between worldview and DAS

Worldview is a concept commonly used in the field of psychotherapy to offer new ways of exploring symptoms of mood disorders, such as DAS, in youth. A worldview is a general perspective on life, society, and institutions, derived from the German word ‘Weltanschauung’ (Wolman, 1973). Understanding people’s worldviews can help explain their behavior (Arrows, 2024; Jones, 1972).

Currently, research on worldviews focuses on conceptualization. Kluckhohn (1956) explored value orientations in different cultural contexts using an anthropological framework that includes philosophical and psychological dimensions of beliefs, values, assumptions, and behaviors. Ibrahim (1984) expanded Kluckhohn’s framework to include five variables for measuring worldview: view of human nature, view of relationships, view of nature, view of time, and view of activity. Janoff-Bulman (1989) applied the concept of worldview to trauma therapy, using the dimensions of world benevolence, fairness, predictability, personal values, and self-control to understand the world. The text concludes with a reference to the worldview of the caseworker. Obasi et al. (2009) conceptualized worldview as cosmology, spirituality, immortality, and communitarianism based on the philosophical concept of worldview. These studies provide diversified perspectives for a deeper understanding of worldviews.

Secondly, worldviews are applied in cross-cultural studies. Scholars aim to understand the worldviews held by different countries and compare them to understand the differences in the behaviors of citizens of different countries (Hofstede, 1984; Ihle et al., 1996; Schwartz and Bilsky, 1990; Williams, 2021). Additionally, scholars have attempted to explore the relationship between different religions and worldviews (Lewis Hall and Hill, 2019). Thirdly, the concept of worldview is applied to psychotherapy (Cummings, 2013; Grieger and Ponterotto, 1995). Psychologists have utilized worldview scales to comprehend the mental health of their clients. For instance, Ibrahim and Heuer (2015) contends that worldview impacts the emotions and actions of an individual. Therefore, it is crucial to assess an individual’s worldview to better understand their mental problems and disorders.

However, there is still a lack of research on worldview. Most scholars have used the Worldview Scale to understand the worldview of their caseworkers, but few have investigated the mechanisms of action between worldview and mental health. Only Chen et al. (2016) found that worldview affects individual mental health through the mediating role of positive self-view. This provides new ideas for better understanding the impact of worldview on DAS in youth populations.

### 1.3 Psychological flexibility as a mediator

Research on psychological flexibility began with American psychologist Hayes et al. (2011), who defined it as an individual’s capacity to mindfully acknowledge and accept their emotions, thoughts, feelings, and memories while remaining committed to pursuing personally meaningful life goals and value. As a foundational concept in Acceptance and Commitment Therapy (ACT), psychological flexibility is developed through six core ACT processes. These processes support individuals in reinterpreting past experiences, maintaining present-moment awareness, and making proactive life choice aligned with their values (Hayes et al., 2006).

Psychological flexibility is particularly pertinent to youth, as its development is substantially influenced by multiple factors, including environmental context, physiological conditions, and levels of self-awareness (Shen, 2019). Existing research on psychological flexibility falls into three main categories. The first category involves studies in which psychological flexibility is used as a measure to predict an individual’s job performance, level of burnout, quality of professional life, and well-being in life (Bond and Bunce, 2003; Garner et al., 2023; Gribbons, 2023; Sauer, 2023). The second category involves studies that combed through applied research on psychological flexibility, in the case study area, where interventions for patients with anorexia nervosa, adolescents with long-term chronic pain disorders, and nonverbal learning disabilities have achieved significant results in helping patients to improve their conditions by enhancing psychological flexibility (Larkin, 2012; Zhu et al., 2020; Wicksell et al., 2009).

The third category explores the role of psychological flexibility as an important variable that is significantly and negatively correlated with anxiety and depressed mood, psychological distress, and psychiatric disorders (Bond and Bunce, 2000; Bond and Bunce, 2003; Bryan et al., 2015; Li et al., 2019), and can alleviate psychiatric disorders and chronic disease-induced pain (Butryn et al., 2015; Zhao et al., 2021), and can mediate the relationship between loneliness and variables such as psychological adjustment, burnout, and mental health (Akdeniz and Gültekin Ahçı, 2023; Chong et al., 2023).

To conclude, some existing studies confirm the relationship between individuals’ perceptions and experiences and psychological flexibility (Hayes et al., 2006; Shen, 2019), while others suggest that psychological flexibility influences youth’s mental health (Bryan et al., 2015; Hayes et al., 2006; Li et al., 2015). In China, studies have confirmed that youth mental health problems threaten the quality of life and life safety of youth and that improving youth psychological flexibility can reduce stress and alleviate anxiety and depression (Xu, 2018; Zhang et al., 2021). Therefore, it is valuable to explore whether youth’s psychological flexibility directly affects their mental health and whether it mediates the relationship between worldview and mental health problems.

### 1.4 The present research

After reviewing existing research, in this study we conclude that further investigation is needed to demonstrate the relationship and mechanism of action between worldview and DAS among youth. The study aims to investigate the relationship between the worldviews and psychological flexibility held by youth and their DAS.

According to previous studies (e.g., Guo et al., 2023; Mifsud and Sammut, 2023; Urbańska-Grosz et al., 2024; Wang et al., 2023), we put forward the following two research hypotheses:

1. The worldviews held by young people can directly predict their DAS.
2. Additionally, the worldviews held by youth can indirectly predict their DAS through psychological flexibility.

**Figure 1:**
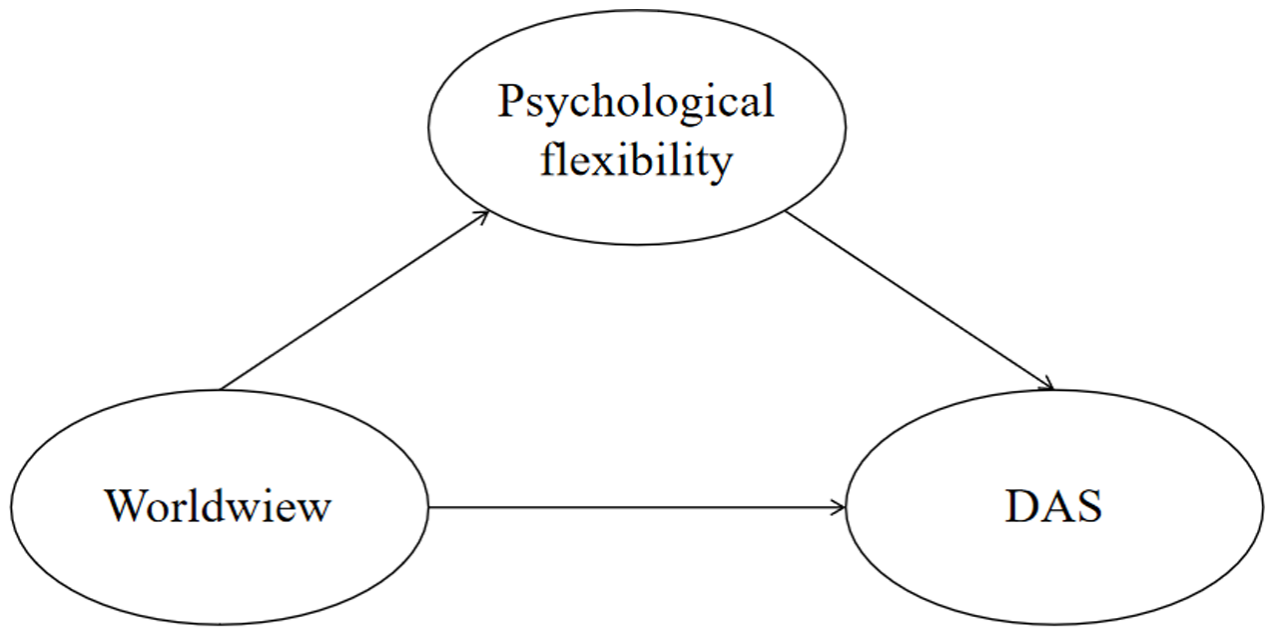
Hypothetical model.

The model proposed in this study is presented in Figure 1.

## 2 Methods

### 2.1 Procedures

From August 27 to October 7, 2023, the research team distributed online questionnaires and managed data through the Questionnaire Star platform. To ensure a diverse sample, the team contacted educational institutions at all levels, including secondary schools and universities in various provinces, and communicated with teachers at each institution. The research team utilized this channel to send the questionnaire link to both current students and graduates of the youth groups, with the assistance of the teachers. The survey questionnaire took approximately 20 min to complete. To encourage more youth groups to participate, we utilized a random red packet reward system. Each participant who completed the questionnaire had a one-quarter chance of receiving a random red packet containing 2-5RMB.

This study received approval from the university’s ethics committee. All participants were informed of the study’s purpose and procedures, provided consent prior to participation, and were assured of data confidentiality and voluntary participation.

### 2.2 Measures

We utilized an online questionnaire comprising a demographic background information statistical form and three scales. The Demographic Background Information Statistical Sheet contained questions aimed at gathering personal information about the respondents, such as gender, age, ethnicity, geographic distribution, political affiliation, household type, and household economic status. Below is a description of the three scales.

#### 2.2.1 Depression-anxiety-stress scale

The Depression-Anxiety-Stress Scale (Antony et al., 1998) is a self-report scale that assesses the respondent’s levels of DAS. It consists of 21 items on a 4-point Likert scale (0 = not at all, 1 = sometimes, 2 = often, 3 = almost always). Studies across different countries have demonstrated that DASS-21 translations maintain reliable psychometric properties, including internal consistency, test–retest reliability, and split-half reliability (Bados et al., 2005; Gong et al., 2010; Moussa et al., 2001; Sardá et al., 2008).

**Figure 2:**
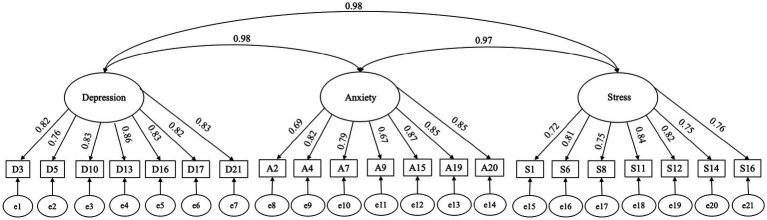
Confirmatory factor analysis of DASS.

The study utilized the Chinese version of the DASS-21 (Gong et al., 2010) to measure three dimensions. The scores of the items in each dimension were summed for scoring: (1) The Depression dimension, which measures the degree of psychological symptoms such as low mood, loss of interest and pleasure, decreased energy, and self-denial (seven items); (2) The Anxiety dimension, which measures the degree of emotional symptoms such as restlessness, nervousness, and worry among the respondents (seven items); and (3) The Stress dimension, which measures the extent to which respondents experience discomfort due to external circumstances or internal demands that exceed the individual’s ability to cope (seven items). The study confirmed the reliability and validity of the DASS-21 through confirmatory factor analysis (*N* = 1,000; see Figure 2). Table 1 displays the Cronbach’s alpha values and model fit. These results demonstrate that the Cronbach’s alpha value, model fit index, and loading coefficients of DASS-21 meet the requirements of psychometric indicators, with relatively good reliability and validity.

**Table 1:** Cronbach’s alpha and model fit of the three scales.

	DASS	SAWV-21	CompACT-17
Cronbach’s alpha	0.97	0.87	0.88
CMIN/DF	5.09	8.01	5.34
RMSEA	0.04	0.06	0.04
CFI	0.99	0.95	0.98
AGFI	0.95	0.93	0.96
IFI	0.99	0.95	0.98
TLI	0.98	0.92	0.97

RMSEA, Root Mean Square Error of Approximation; CFI, Comparative Fit index; AGFI, Adjusted Goodness-of-fit index; IFI, Incremental Fit index; TLI, Tucker-Lewis index.

#### 2.2.2 Scale to assess world views

The Scale to Assess World Views (Ibrahim and Heuer, 2016) is a self-report scale that consists of 30 items on a 5-point Likert scale (1 = strongly disagree, 2 = disagree, 3 = neutral, 4 = agree, and 5 = strongly agree). It examines the participant’s general or standardized human nature, social relationships, nature, time, and activity concepts, and measures four different worldview tendencies: positivism, traditionalism, here-and-nowism, and pessimistic tendencies.

The scale used in the study was originally in English, then translated into Chinese, and finally back-translated into English by the research team in China. The scale comprises four dimensions, and the scores of the items in each dimension are summed for scoring: (1) Positive worldview, a type of worldview in which the individual believes in the intrinsic goodness of human beings, their willpower and agency, and emphasizes that human activities should focus on both internal growth and external development (nine items). An example item is, “If we have enough willpower, no disadvantage or difficulty can stop us from moving forward”; (2) Traditional worldviews, which emphasize social hierarchy, respect for tradition and authority, and the belief that nature can be controlled (eight items); (3) Spontaneous worldviews, which emphasize experiencing life in the present moment or ‘in the here and now’ and prioritize relaxation and enjoyment (six items); and (4) Pessimistic worldviews, in which the individual believes that human beings are evil and self-interested by nature and that they are powerless in the face of social and natural forces (seven items). An example item is, “Human nature is such that there will always be wars and conflicts.”

To verify the structural validity of the scale, an exploratory factor analysis (*N* = 1,351) was conducted to screen its items. The results indicated that the KMO (Kaiser-Meyer-Olkin) was 0.95 and Bartlett sig was less than 0.05, meeting the criteria for conducting factor analysis (Kaiser, 1974) and making it suitable for EFA. The rotated component matrix indicates that the scale still comprises four dimensions: positive worldview, traditional worldview, spontaneous worldview, and pessimistic worldview. However, the four dimensions now contain different items than those in the original scale. For instance, the item “I feel rather powerless in the face of the forces of nature,” which was originally categorized under the pessimistic worldview, is now classified under the spontaneous worldview. Additionally, the study removed nine items that had loading coefficients lower than the dimension average and were not applicable in the Chinese context. Therefore, the revised scale (SAWV-21) has 21 items, with seven items each in the positive and traditional worldview dimensions, three items in the spontaneous worldview dimension, and 4 items in the pessimistic worldview dimension.

**Figure 3:**
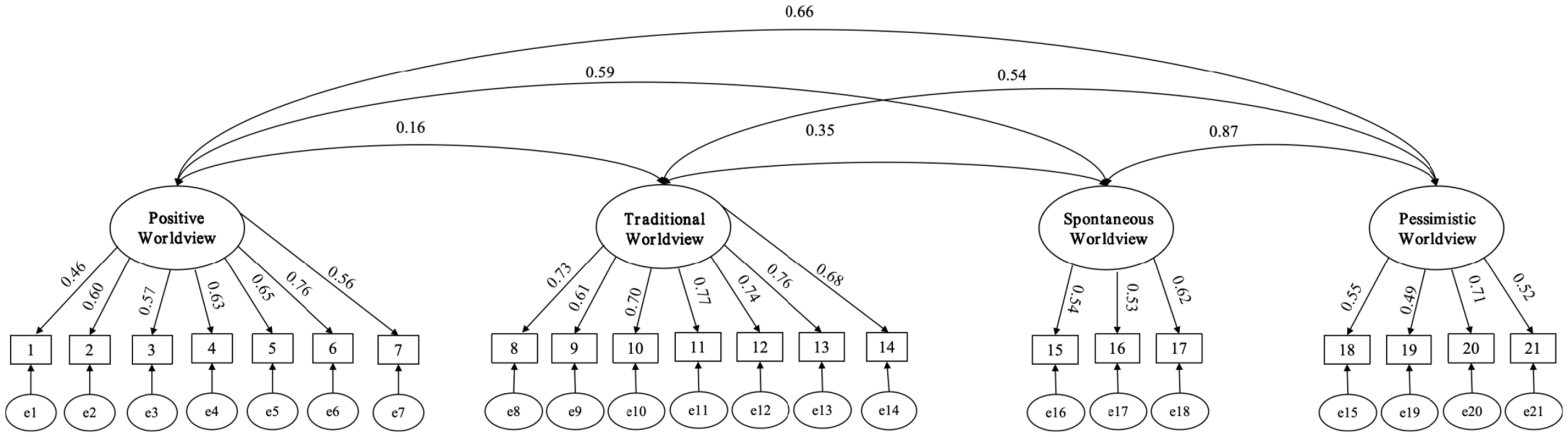
Confirmatory factor analysis of SAWV-21.

The study confirmed the reliability and validity of the SAWV-21 using confirmatory factor analysis (*N* = 1,000). The results, presented in Figure 3 and Table 1, demonstrate that the Cronbach’s alpha value, model fit indices, and loading coefficients of the SAWV-21 meet the requirements of psychometric indicators, indicating good reliability and validity with fewer items in comparison to Ibrahim’s scale.

#### 2.2.3 CompACT scale

**Figure 4:**
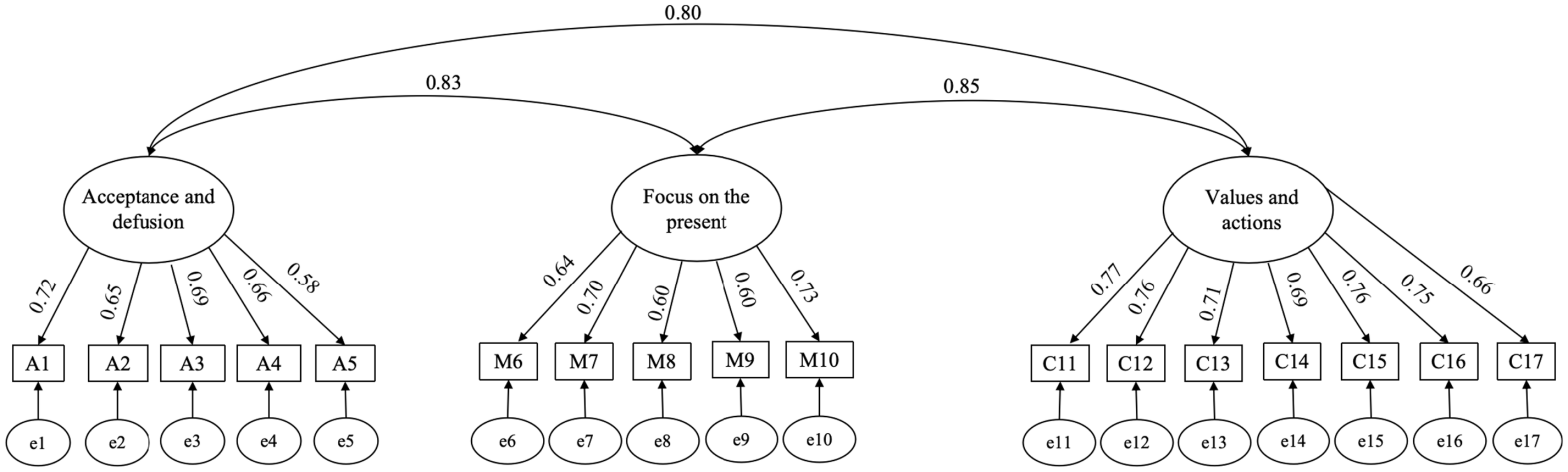
Confirmatory factor analysis of CompACT.

The CompACT Scale was initially developed by Francis et al. (2016) to comprehensively measure respondents’ psychological flexibility. Since then, it has been translated and adapted into various versions. The self-report Chinese version of the CompACT Scale used in this study is measured on a 5-point Likert scale (1 = strongly disagree, 2 = disagree, 3 = neutral, 4 = agree, and 5 = strongly agree). It was developed based on the CompACT-15 Scale by Fang and Huang (2023) and authorized for use by the Chinese Academy of Sciences (CAS). Up to now, there is no published literature related to this scale. The scale consists of three dimensions and 17 items, which are scored by summing the scores of the items in each dimension, with the acceptance and defusion dimensions requiring reverse scoring: (1) The Acceptance and Defusion dimension, which measures the extent to which the respondent actively confronts, accepts, and defuses ideas from personal experiences (five items); (2) The Focus on the Present dimension, which measures the degree to which one focuses on the present moment and is aware of one’s thoughts (five items); and (3) The Values and Actions dimension, which measures the extent to which respondents know what they want and can act on it effectively (seven items). The study confirmed the reliability and validity of the CompACT Scale through confirmatory factor analysis (*N* = 1,000). The results are displayed in Figure 4 and Table 1, which meet the requirements of psychometric indicators, demonstrating good reliability and validity.

### 2.3 Data analysis

The data was analyzed using SPSS 27.0 and Amos 26.0. As the survey was conducted online and only complete answers were submitted, there were no missing values. Extreme values were processed using SPSS 27.0 by removing 1% of the extreme values in different dimensions of the DASS, SAWV-21, and CompACT scales by shrinking the respective tails and removing a total of 114 samples. This resulted in a final sample size of 2,237. The study tested for correlation and found no high correlation (|*r*| < 0.80) between the independent variables. The results of multiple covariance statistics showed that the variance inflation factor (VIF) and tolerance of the independent variables met the statistical criteria, indicating no covariance problem in the independent variables.

Exploratory and confirmatory factor analyses were conducted on the SAWV-30 and developed SAWV-21 scales, as well as the other two scales, respectively, using SPSS 27.0 and AMOS 26.0. The current stage of DAS, worldviews, and psychological flexibility among youth was explored by describing each subscale of the three scales using SPSS 27.0.

The study utilized Structural Equation Modeling (SEM) to assess the validity of the hypothesized model (refer to Figure 1). SEM is a statistical analysis technique that examines and confirms intricate relationships between variables, considering both direct and indirect effects between observed data and latent variables (Jacobucci et al., 2016). In this study, DAS were treated as latent variables with corresponding explicit variables as subscales. However, the loading coefficients between psychological flexibility and its three subscales were low. It is important to note that through this study we aimed to investigate the impact and mechanisms of different worldviews on DAS among youth. The study re-established an SEM by adding the three dimensions of psychological flexibility as observable variables, in addition to the four dimensions of youth’s worldviews as observed independent variables.

## 3 Results

### 3.1 Participants

The research team collected 2,357 valid questionnaires that were filled in by young people who were aged between 15 and 24. Six outliers with the same scores in all four dimensions were removed, resulting in a final sample size of 2,351 people. Table 2 shows the types and value intervals of the demographic background variables of the respondents. The youth that completed the questionnaire had a relatively balanced gender distribution, with 1,159 males (49.7%) and 1,192 females (50.7%). The majority of the participants were aged 18–24, accounting for 78.6% of the total; the other 502 participants were aged 15–18, accounting for 21.4%. Han Chinese made up the majority of the ethnic distribution, totaling 2,157 people, accounting for 91.7%. The current status of contemporary Chinese youth’s worldview, psychological flexibility, and their DAS is as shown in Table 3.

**Table 2:** Participant descriptive statistics.

Variables	*n*	%
Gender		
Male	1,159	49.3%
Female	1,192	50.7%
Age		
15–17	502	21.4%
18–24	1,949	78.6%
Ethnicity		
Han	2,157	91.7%
other	194	8.3%
Geographical distribution		
Large city	636	27.0%
Medium-sized city	719	30.6%
Small town	535	22.8%
Countryside	461	19.6%
Political outlook		
Mass	910	38.7%
Communist youth league member	1,101	46.8%
Democratic party member	83	3.6%
Party member	257	10.9%
Account type		
Rural household	838	35.6%
Urban household	586	24.9%
Resident household (previously agricultural)	340	14.5%
Resident household (previously urban)	328	14.0%
Only child or not		
Yes	1,122	47.72%
No	1,229	52.28%
Family economic status		
Lowest	117	5.0%
Medium–low	674	28.7%
Medium	1,023	43.5%
Medium–high	363	15.4%
Highest	174	7.4%

**Table 3:** Descriptive statistics for worldview, psychological flexibility and psychological condition.

	*N*	*M*	Sd	Df	*T*	*p*
POWV	2,237	4.08	0.57	2,236	89.27	<0.001
TWV	2,237	2.94	0.97	2,236	−3.16	<0.01
SWV	2,237	3.73	0.77	2,236	45.02	<0.001
PEWV	2,237	3.60	0.70	2,236	40.46	<0.001
ACT	2,237	3.68	0.55	2,236	58.45	<0.001
ST	2,237	2.23	0.81	2,236	42.75	<0.001
AN	2,237	2.13	0.82	2,236	36.37	<0.001
DE	2,237	2.05	0.87	2,236	30.00	<0.001

POWV, positive worldview; TWV, traditional worldview; SWV, spontaneous worldview; PEWV, pessimistic worldview; ACT, psychological flexibility; ST, stress; AN, anxiety; DE, depression.

### 3.2 SEM results

**Figure 5:**
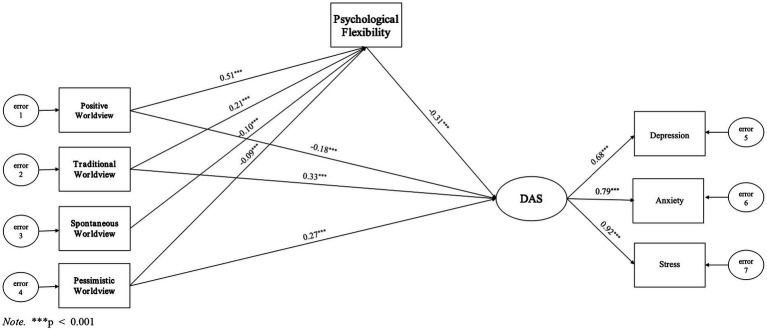
Structural equation model path diagram of the interrelations between worldview, psychological flexibility, and DAS. ***p < 0.001.

The model fit indices demonstrate the adequacy of the proposed model, with *X*^2^/df = 3.813, RMSEA = 0.035, GFI = 0.998, AGFI = 0.985, CFI = 0.999, IFI = 0.999, and TLI = 0.993, all of which are at an excellent level. Furthermore, the study utilized Amos 28.0 to explore the model and removed the insignificant direct covariate path between spontaneous worldview and DAS. The final model results are presented in Figure 5. The standardized loadings of the latent variables in the model ranged from 0.68 to 0.92 for DAS, all exceeding the critical value of 0.50, indicating that the observed subscales adequately measured the latent variables.

#### 3.2.1 Direct predictive effect

Table 4 presents the SEM results, indicating that positive worldview had a direct negative predictive effect on DAS with an effect size of −0.18. Additionally, it had an indirect negative predictive effect on DAS, which was mediated by psychological flexibility with an effect size of 0.51* to −0.31 = −0.16. Overall, the total predictive effect of positive worldview on DAS was negative with an effect size of −0.18 to −0.16 = −0.34. The traditional worldview had a direct positive predictive effect on DAS with an effect size of 0.33, as well as an indirect negative predictive effect on DAS mediated by psychological flexibility, with an effect size of 0.21* to −0.31 = −0.07. Overall, the total predictive effect of the traditional worldview on DAS was positive, with an effect size of 0.33–0.07 = 0.26.

**Table 4:** Results of structural equation model analysis.

Model	POWV	TWV	SWV	PEWV	ACT
Direct effects					
DAS	−0.18***	0.33***		0.27***	−0.31***
Indirect effects					
ACT	0.51***	0.21***	−0.10***	−0.09***	
DAS	−0.16***	−0.07***	0.03***	0.03***	
Total					
DAS	−0.34***	0.26***	0.03***	0.30***	

POWV, positive worldview; TWV, traditional worldview; SWV, spontaneous worldview; PEWV, pessimistic worldview; ACT, psychological flexibility; DAS, depression, anxiety, stress.

***p < 0.001.

#### 3.2.2 Indirect predictive effect

In contrast, psychological flexibility fully mediated the relationship between spontaneous worldview and DAS. This suggests an indirect positive effect of spontaneous worldview on DAS mediated by psychological flexibility, with an effect size of −0.10* to −0.31 = 0.03. Pessimistic worldview had a direct positive effect on DAS with an effect size of 0.27, and an indirect positive predictive effect on DAS mediated by psychological flexibility with an effect size of −0.09* to −0.31 = 0.03. Taken together, the results indicate a positive total predictive effect of a pessimistic worldview on DAS, with an effect size of 0.30.

Overall, the results of the model support hypotheses 1 and 2. We found that the type of worldview held by Chinese youth, mediated by psychological flexibility, directly and indirectly, predicts their level of DAS. Specifically, a positive worldview had a direct negative effect on DAS, while traditional and pessimistic worldviews had a direct positive effect on DAS. All four dimensions of worldview had indirect effects on DAS through psychological flexibility. Positive and traditional worldviews negatively correlated with DAS, while spontaneous and pessimistic worldviews positively correlated with DAS.

## 4 Discussion

This study provides a detailed analysis of the direct and indirect effects of worldview on mental health problems among contemporary Chinese youth, with psychological flexibility serving as a mediator. These findings contribute to our understanding of the associations between worldview, psychological flexibility, and mental health, offering insights into how various worldviews influence youth mental health outcomes in the context of a rapidly changing society.

### 4.1 Predictive effects of worldview on DAS

Our findings indicate that a positive worldview has a negative predictive effect on mental health problems, suggesting that such a worldview promotes mental well-being. This aligns with prior research demonstrating that positive perspectives support resilience and mitigate mental health risks (Chen et al., 2009; Cummings, 2013; Jecmen, 1993; Sue, 1977). Conversely, the traditional worldview exerts a positive effect on mental health problems, in line with studies showing that adherence to traditional beliefs can increase psychological distress, particularly among youth adapting to a rapidly evolving world (King et al., 2022; Koesten et al., 2009; Porter, 2012). This correlation highlights the need to critically assess the impact of traditional values on mental well-being, especially in dynamic sociocultural contexts where traditionalism may hinder adaptability and openness to change. Similarly, a pessimistic worldview has a strong positive effect on DAS, exacerbating the frequency and severity of mental problems. Pessimism is often characterized by negative perspectives on life and low expectations for the future, which can increase and decrease perceived social support, contributing to mental health problems such as low self-worth, a diminished sense of belonging, and even suicidal ideation (Chen et al., 2016; Lam et al., 2010; Mitchelson and Burns, 1998; Smith et al., 1988).

### 4.2 Mediating effects of psychological flexibility

The SEM results suggest that psychological flexibility plays a key mediating role in the relationship between worldview and DAS, indicating that flexibility may be a valuable target in mental health interventions. Increased psychological flexibility was associated with reduced mental health problems among youth, aligning with previous findings that higher flexibility correlates with better emotional regulation (Bond and Bunce, 2001; Bond and Bunce, 2003; Bryan et al., 2015; Li et al., 2019; Wicksell et al., 2009).

The study further demonstrates that a positive worldview indirectly reduces DAS through enhanced psychological flexibility. Youth with a positive worldview appear more adept at flexible thinking, reinterpreting internal conflicts, experiencing positive emotions, and fostering self-efficacy, which collectively improve in psychological flexibility (Hayes et al., 2006; Shen, 2019). This suggests that cultivating a positive outlook may serve as a practical approach to improve youth adaptability, thereby reducing symptoms of DAS (Bryan et al., 2015; Li et al., 2015). At the same time, the traditional worldview indirectly reduces DAS through enhanced psychological flexibility. This indirect effect is validated for the first time and is inconsistent with the direction of the previously observed direct effect (King et al., 2022; Koesten et al., 2009; Porter, 2012), which may be related to the role of a traditional worldview in providing stable values, coping with change, and accommodating new ideas.

Psychological flexibility fully mediates the indirect positive predictive effect of the spontaneous worldview on youth’s mental health problems. Although spontaneity may enhance present-moment focus, it can reduce adaptability if accompanied by excessive self-focus and insensitivity to external feedback (Höping et al., 2006; Schwartz-Mette and Rose, 2009). This may impair emotional regulation, increasing the risk of mental health problems. Finally, a pessimistic worldview has an indirect positive predictive effect on DAS mediated by psychological flexibility. This aligns with research showing that pessimism is frequently linked with negative coping mechanisms, such as avoidance and withdrawal, which weaken cognitive and emotional regulation (Bakan, 1966; Blatt and Blass, 1992; Chen et al., 2016; Thompson and Zuroff, 2004). This reduced adaptability hinders their ability to cope effectively with stressors, emphasizing the importance of addressing pessimistic worldviews in interventions aimed at enhancing youth mental health.

Overall, by framing worldview and psychological flexibility as interconnected influences on DAS, our findings offer valuable insights for interventions that target youth mental adaptability in response to societal changes. Notably, recent research suggests that post-COVID-19, there may be a trend of rising anxiety, depression, and stress levels among youth (Beal, 2022; Carey et al., 2022). Our findings offer new perspectives on enhancing youth mental health through worldview development. Specifically, forming a positive worldview may help foster psychological flexibility, potentially reducing psychological burdens associated with more negative or pessimistic perspectives.

## 5 Significance, limitations, and implications

In this study, we examine the relationship between the worldviews of youth and their mental health status, mediated by psychological flexibility, in the context of the rapid development of information technology. The focus is on the youth cohort at a critical stage of growth and development. One aim of the study is to contribute to existing research on youth mental health in China by investigating the causes and mechanisms of youth mental problems. Secondly, this study introduces the concept of ‘worldview’ to the study and discussion of youth mental health problems, providing new perspectives. The SAWV-21 was revised and developed to improve the applicability of the Worldview Scale in China and promote its localization through the collection and analysis of Chinese youth worldview data. Finally, this study provides evidence suggesting a mediating role for psychological flexibility. Improving psychological flexibility can effectively reduce the level of DAS in youth; more specifically, it enhances the positive effects of positive worldview on DAS and mitigates the negative effects of spontaneous worldview, traditional worldview, and pessimistic worldview on DAS. In this study, we examine the direct and indirect effects of individual worldviews and psychological resources on DAS, contributing to research on psychological flexibility in youth.

However, this study has certain limitations. We employed a combination of stratified and random sampling for data collection; however, this approach may have introduced sample selection bias, as certain groups might be overrepresented, potentially impacting the data’s representativeness. Additionally, the study used cross-sectional data, so future research could explore the mechanism of action between youth worldviews and DAS through a longitudinal research design.

The study’s findings offer valuable insights for youth populations worldwide seeking to alleviate DAS. By examining various worldviews instead of treating worldview as a single variable, we can more effectively investigate the impact and mechanisms of worldviews on mental health problems among young people. This, in turn, allows for more targeted guidance and recommendations for shaping and guiding youth’s worldviews.

The government and society should prioritize the cultivation and shaping of young people’s worldview and develop education programs aimed at alleviating mental health problems. Specifically, holding a positive worldview can effectively alleviate mental health problems among young people, and at a time of high incidence of psychological problems among young people, education on a positive view of human nature should be carried out to build up young people’s basic confidence in human nature and their hope for the fate of mankind, and to guide young people to form a worldview inclination that is positive, harmonious and kind (Chen et al., 2016; Sears, 1986). Secondly, a pessimistic worldview implies negativity, which in itself is negative for youth groups and can exacerbate such emotions as stress, anxiety, and depression among youth. Therefore, attention to and intervention in young people’s bad moods and negative perceptions should be strengthened, and action should be taken to provide help and support for young people’s life, study, and work, to build a youth-friendly social environment, to create a caring and tolerant atmosphere for young people in society as a whole, to alleviate the challenges and pressures imposed on young people by the times, to ease their psychological burdens, and to guide young people to form a tendency toward a worldview conducive to the development of their physical and mental well-being (Cheung et al., 2022; Kewley et al., 2018). Finally, the impact of traditional and spontaneous worldviews on the mental health of young people is complex, with a negative impact on youth mental health, but also containing a stabilizing aspect and the youth cohort’s concern for and enjoyment of life in the present, which should be viewed dialectically, with sufficient attention, appropriate guidance, and intervention (Arnold et al., 2016; Lertzman, 2002).

The findings indicate that psychological flexibility may not only promote mental health, but also mediate the influence of worldview on mental health problems. Specifically, psychological flexibility appears to strengthen the positive effects of a positive worldview on mental health, and mitigate the adverse effects of a traditional worldview. This provides new opportunities for exploring and attempting interventions and treatments for youth mental health problems. High psychological flexibility allows individuals to quickly adapt their behavior and mindset to their life circumstances. This enables them to better handle the challenges and pressures of life and make more meaningful lifestyle choices, which is particularly beneficial for young people (Bryan et al., 2015; Hayes et al., 2006; Li et al., 2015).

Therefore, when conducting psychological interventions for youth groups, it is important to recognize the significant role that psychological flexibility plays in individual mental health. Attention should be given to cultivating and enhancing youth psychological flexibility, which has six core elements: acceptance, defusion, self as context, contact with the present moment, values, and committed action (Hayes et al., 2011). So it is important to teach and train youth groups to be more open to their feelings, be more defused from their cognition, be more mindful, increasingly take the perspective of self-as-context, clarify their values more, and be more committed to actions that are in line with their values. This can help them choose a more valuable and fulfilling lifestyle, which is beneficial for young people. Youth groups should be encouraged to take initiative and enhance their active constructive awareness. They should learn and apply techniques such as positive thinking, meditation, cognitive defusion, and committed action to improve their psychological flexibility (Baer, 2003; Hayes et al., 2006; Shen, 2019; Teasdale et al., 2000).

## Data Availability

The raw data supporting the conclusions of this article will be made available by the authors without undue reservation.
